# Factors Influencing Maternal Healthcare Experiences Among Black African Women in the United Kingdom: A Systematic Review Protocol

**DOI:** 10.1002/hsr2.73450

**Published:** 2026-10-28

**Authors:** Irene Devine Dzirasa, Nasima Akhter, Esther Mireku, Modestus Okonkwo, Lawrence Achilles Nnyanzi

**Affiliations:** ^1^ Teesside University Middlesbrough UK; ^2^ Durham University Durham UK; ^3^ North Tees and Hartlepool NHS Foundation Trust Stockton‐on‐Tees UK; ^4^ Seeta University Mbalala Mukono Uganda

**Keywords:** antenatal care, barriers, Black African women, childbirth, experiences, facilitators, intrapartum care, maternal healthcare, postnatal care, United Kingdom

## Abstract

**Background and Aims:**

Understanding the experiences of Black African women seeking and receiving maternal healthcare in the United Kingdom is crucial because cultural and ethnic backgrounds may shape health‐seeking behaviors and interactions with the healthcare system. Synthesizing existing qualitative evidence on the factors influencing maternal healthcare experiences among Black African women in the United Kingdom is important for informing high‐quality, culturally appropriate interventions and policies. This review aims to synthesize qualitative literature to explore the nature and significance of factors influencing maternal healthcare experiences among Black African women in the United Kingdom. It will identify barriers and facilitators, as well as wider determinants of maternal healthcare experiences. The review will also explore potential interventions that may improve experiences and outcomes during pregnancy, childbirth, and the postnatal period. Ultimately, the review will identify implications for culturally sensitive and responsive maternal health services that address the needs of Black African women in the United Kingdom.

**Methods:**

The review will explore factors influencing maternal healthcare experiences among Black African women in the United Kingdom. Relevant studies will be identified through MEDLINE, CINAHL, and EMBASE, along with grey literature searches, using a predefined search strategy. The Critical Appraisal Skills Programme (CASP) checklist will be used to appraise the methodological quality of included studies. Data from included studies will be synthesized using thematic synthesis.

**Conclusion:**

The systematic review will support policymakers, maternal healthcare providers, and researchers in understanding Black African women's experiences of, and barriers to, maternal healthcare in the United Kingdom. It will also synthesize evidence on factors that facilitate access to and positive experiences of maternal healthcare among Black African women in the United Kingdom.

**Systematic Review Registration:**

The review has been registered with the International Prospective Register of Systematic Reviews (PROSPERO) (CRD420251007412).

**Abbreviations**
CASP: Critical Appraisal Skills Programme
JBI: Joanna Briggs Institute
MeSH: medical subject headings
NHS: National Health Service
PRISMA: Preferred Reporting Items for Systematic Reviews and Meta‐Analyses
PRISMA‐P: Preferred Reporting Items for Systematic Review and Meta‐Analysis Protocols
PROSPERO: International Prospective Register of Systematic Reviews

## 1 Background

In the United Kingdom, maternal healthcare is a core component of the National Health Service (NHS), and pregnant women are entitled to maternity care irrespective of immigration status or ability to pay [1]. This provision is intended to support access to essential services throughout pregnancy, childbirth, and the postnatal period. NHS maternity services include routine antenatal appointments, diagnostic screening, midwifery and obstetric care, and specialized support where complications arise [2].

Despite this entitlement, disparities in the quality, accessibility, and outcomes of maternity care persist. Assessments of maternity services have identified concerns including staffing pressures, communication problems, and reduced confidence in care provision [3, 4]. These concerns have not affected all demographic groups equally.

Black women in the United Kingdom experience a disproportionately high risk of adverse maternal outcomes, including higher rates of maternal mortality than White women [5, 6]. These inequalities are associated with interacting clinical, socioeconomic, and structural factors. Research also indicates that Black African women may encounter multiple barriers that affect access to and experiences of maternity care [7].

Socioeconomic disadvantage can influence access to healthcare. Financial insecurity, employment conditions, transport costs, and difficulty taking time off work may affect attendance at appointments [8, 9]. For some migrant women, uncertainty about entitlement to care and limited information about eligibility or available support may further discourage timely access to services [10].

Cultural and linguistic factors can also shape maternal healthcare experiences. Cultural beliefs and practices relating to childbirth, postnatal care, and diet may not always align with standard service provision, and women may feel that their needs are overlooked [11, 12]. Language barriers can impede communication, making it more difficult to express concerns or understand clinical information and advice [13].

Structural and interpersonal racism may also adversely affect maternity care experiences. Reports and qualitative studies describe racial stereotyping, implicit bias, and women feeling that their pain or symptoms are dismissed [14]. Such experiences may contribute to mistrust and feelings of disempowerment within healthcare settings [12].

Several factors may facilitate access to and positive experiences of maternal healthcare among Black African women. Culturally responsive care may help address some of these barriers by recognizing relevant cultural practices, responding respectfully to individual preferences, and ensuring access to appropriate interpretation support [15, 16].

Community engagement may also facilitate access to care. Community networks and trusted advocates can provide information, practical guidance, and emotional support to help women navigate healthcare services [8, 17]. Policies that address structural inequalities and promote inclusion may also support more equitable access [17, 18]. Family and social support can provide additional practical and emotional assistance, particularly where women experience language barriers or unfamiliarity with healthcare services [19, 20].

A trusting patient−provider relationship characterized by empathy, respect, and effective communication may influence women's experiences of care and engagement with services [21, 22].

Although there is growing interest in maternal health inequalities affecting Black women, existing systematic reviews do not sufficiently isolate the maternal healthcare experiences of Black African women in the United Kingdom. Black African women are often included within broader categories such as “ethnic minority,” “Black,” or “migrant” women, which can obscure culturally specific, migration‐related, and structural factors. This review, therefore, focuses specifically on Black African women in the United Kingdom.

Onanuga et al., in “Equity in reproductive health: A systematic review of factors influencing higher maternal mortality among Black women in the United Kingdom,” focused primarily on factors associated with maternal mortality among Black women. The review did not disaggregate Black African and Black Caribbean women, limiting its ability to examine factors that may be specific to Black African women [23].

Toh and Shorey, in “Experiences and needs of women from ethnic minorities in maternity healthcare,” synthesized qualitative studies involving a range of ethnic minority groups. Although the review identified themes including communication barriers and cultural insensitivity, findings were aggregated across diverse populations, limiting conclusions that can be drawn specifically about Black African women in the United Kingdom [24].

These reviews demonstrate important ethnic inequalities in maternal health but provide a limited population‐specific synthesis for Black African women. The proposed review will address this gap by examining how factors such as migration experiences, language, culture, social support, and healthcare‐system interactions shape maternal healthcare experiences among Black African women in the United Kingdom.

## 2 Methods

### 2.1 Protocol and Registration

This protocol has been developed in accordance with the Preferred Reporting Items for Systematic Review and Meta‐Analysis Protocols (PRISMA‐P) 2015 guidance [25, 26, 27]. The review has been registered with the International Prospective Register of Systematic Reviews (PROSPERO; CRD420251007412). It will explore factors influencing maternal healthcare experiences among Black African women in the United Kingdom, including barriers and facilitators to accessing and receiving care.

### 2.2 Eligibility Criteria

This systematic review will include studies involving Black African women living in the United Kingdom who have accessed maternal healthcare services. The UK context is relevant because maternity care is delivered within the NHS, while marked ethnic inequalities in maternal outcomes and experiences persist [28, 29].

National surveillance and related evidence have documented persistent inequalities in maternal outcomes among Black women in the United Kingdom, supporting the need for focused investigation of Black African women's experiences of maternity care [29].

Eligible studies must explore factors influencing maternal healthcare experiences from the perspectives of Black African women. Relevant factors may include barriers, facilitators, cultural influences, structural or systemic factors, and interactions with healthcare professionals and services. Qualitative studies and mixed‐methods studies with extractable qualitative data will be included. The review prioritizes qualitative evidence because it can provide contextualized accounts of how interpersonal, cultural, and systemic factors shape maternal healthcare experiences.

Studies must:

- Clearly define the study population as including Black African women living in the United Kingdom.
- Focus on experiences related to any stage of maternal healthcare (antenatal, intrapartum, or postnatal).
- Be published between 2015 and 2025. This period was selected to capture contemporary UK maternity‐care policy and practice, including major developments in maternity services and immigration‐related healthcare policy during the decade.
- Be published in English, as the review team is proficient in this language.

This review will exclude:

- Studies not conducted in the United Kingdom.
- Studies that include multiple ethnic groups but do not report findings for Black African women separately or in an extractable form.
- Commentaries, editorials, conference abstracts or proceedings, systematic reviews, and purely quantitative studies.

Supporting Information S1: Appendix Table I presents the inclusion and exclusion criteria according to population, phenomenon of interest, study design, publication type, language, geographical scope, and relevant outcomes.

### 2.3 Information Sources and Search Strategy

A comprehensive literature search will be undertaken to identify evidence on factors influencing maternal healthcare experiences among Black African women in the United Kingdom. MEDLINE, CINAHL, and EMBASE will be searched using database‐specific controlled vocabulary and free‐text terms. Grey literature will also be searched, and the reference lists of relevant reviews and included studies will be checked for additional eligible records. Two reviewers (I.D.D. and M.O.) will conduct the searches independently. Search concepts will include terms relating to “Black African women,” “maternal healthcare,” “antenatal care,” “intrapartum care,” “postnatal care,” “experiences,” “barriers,” “facilitators,” and the “United Kingdom.” Terms will be combined using the Boolean operators AND and OR, with Medical Subject Headings (MeSH) and database‐specific subject headings used where appropriate. Truncation (*) will be applied where appropriate to capture word variants. Supporting Information S1: Appendix Table II presents the complete search strings for each database.

### 2.4 Study Selection

Rayyan will be used to manage records retrieved from the electronic searches [30, 31, 32]. After duplicate records have been removed, two reviewers (I.D.D. and M.O.) will independently screen titles and abstracts against the predefined eligibility criteria. Potentially eligible reports will then undergo full‐text assessment. The screening process will be piloted using the eligibility criteria and a predefined screening form to support consistency between reviewers.

The reviewers will record reasons for exclusion at the full‐text stage. Disagreements will be resolved through discussion and, where necessary, consultation with a third reviewer. The planned study‐selection process is illustrated using a PRISMA 2020 flow diagram (Figure 1) [27]. Studies meeting the eligibility criteria will proceed to methodological quality assessment and data extraction.

**Figure 1:**
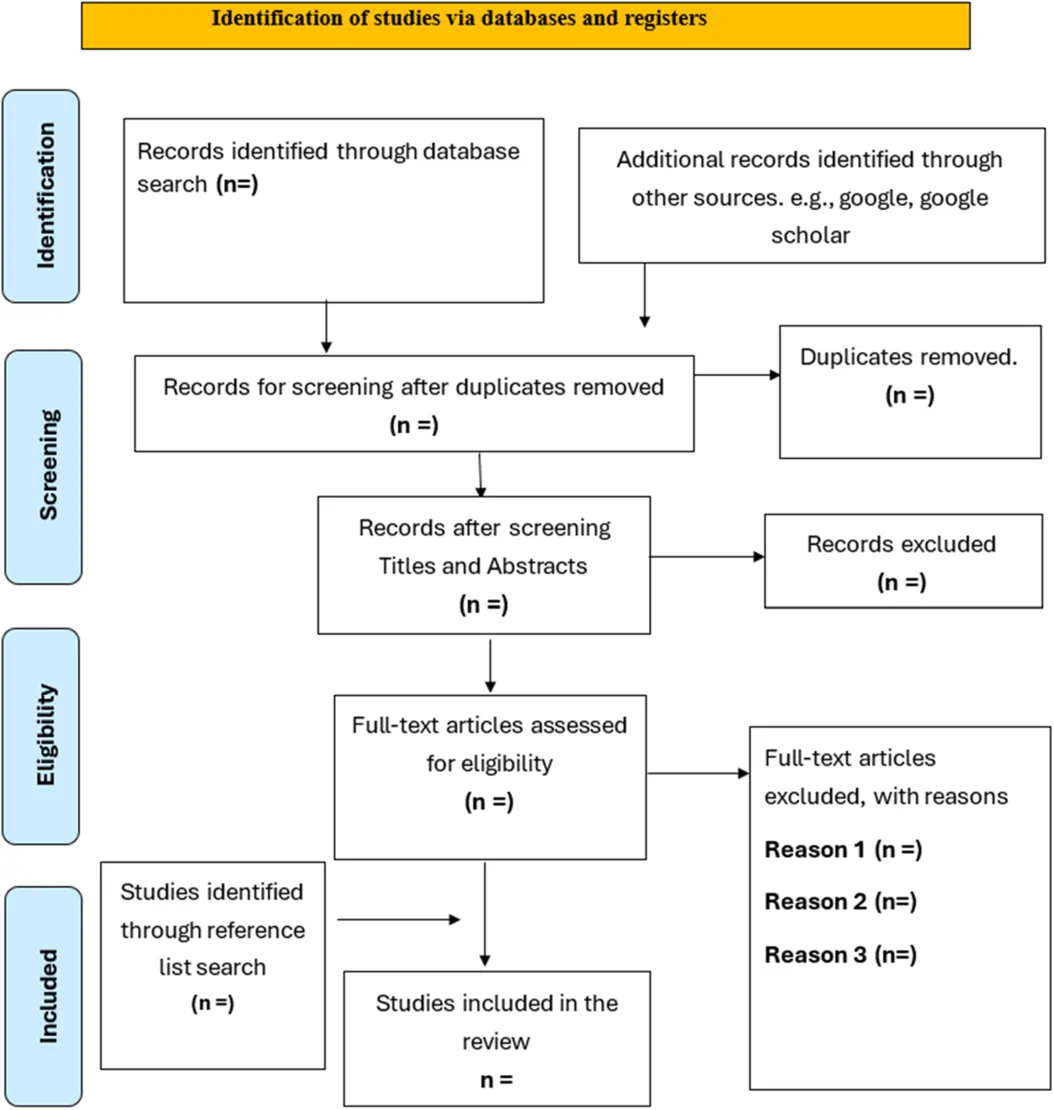
Proposed PRISMA 2020 flow diagram illustrating the planned study selection process for this systematic review. The boxes are placeholders and do not represent completed screening results. Figure adapted from Page et al. [27].

### 2.5 Data Extraction

Two independent reviewers (I.D.D. and M.O.) will use an adapted Joanna Briggs Institute (JBI) data‐extraction form to extract data from eligible studies [33]. The reviewers' extraction forms will be compared, and discrepancies will be resolved through discussion or consultation with a third reviewer where necessary. Supporting Information S1: Appendix Table III presents the preliminary data‐extraction form. Data items will include article title, author(s), year of publication, country and setting, date of extraction, reviewer, study aim, study design, participant characteristics, and findings relating to maternal healthcare experiences, barriers, facilitators, and study limitations.

### 2.6 Quality Assessment of Included Studies

The methodological quality of included qualitative studies will be appraised using the Critical Appraisal Skills Programme (CASP) qualitative checklist [34]. Supporting Information S1: Appendix Table IV presents the checklist. The CASP tool considers domains including the clarity of the research aims, appropriateness of the methodology and design, recruitment, data collection, researcher−participant relationships, ethical considerations, data analysis, clarity of findings, and the value of the research. Two reviewers (I.D.D. and M.O.) will undertake appraisal independently, with disagreements resolved through discussion or consultation with a third reviewer (L.A.N.). Appraisal findings will be reported transparently and used to inform the interpretation of the synthesis rather than to generate an unsupported numerical quality score.

### 2.7 Synthesis of Included Studies

A narrative summary will first describe the characteristics of included studies, including authors, year of publication, study aim, methods, sampling strategy, participant characteristics, and key findings. Qualitative findings will then be synthesized using the thematic synthesis approach described by Thomas and Harden [35].

Reviewers will become familiar with the extracted qualitative findings before undertaking inductive, line‐by‐line coding. Codes will be compared across studies to identify similarities, differences, and recurring concepts [35].

The thematic synthesis will proceed through three stages: (i) line‐by‐line coding of relevant qualitative findings, (ii) organization of related codes into descriptive themes, and (iii) development of analytical themes that extend beyond the descriptive findings to address the review question [35, 36].

Initial coding will be undertaken by I.D.D. in consultation with M.O. I.D.D. and M.O. will then examine relationships among codes and descriptive themes and develop the analytical themes and subthemes. Interpretive decisions will be documented to support transparency, and the final synthesis will identify factors shaping maternal healthcare experiences among Black African women in the United Kingdom.

## 3 Discussion

A better understanding is needed of the barriers, facilitators, and wider factors influencing maternal healthcare experiences among Black African women in the United Kingdom. This protocol sets out the methods for a systematic review that will synthesize qualitative evidence on how these factors shape women's interactions with maternity services.

The review will examine experiences relating to discrimination, fear of mistreatment, language barriers, cultural responsiveness, trust, communication, social support, and access to services. It will also identify factors associated with more positive experiences of care.

By synthesizing these factors, the review may inform recommendations for improving the responsiveness of maternal healthcare services to the needs of Black African women in the United Kingdom. Findings will be disseminated through academic and professional channels, including workshops, seminars, conferences, and publications, to inform policy, practice, and future research.

### 3.1 Limitations

Potential limitations include restriction to English‐language studies conducted in the United Kingdom, exclusion of purely quantitative studies, and limited ability to examine heterogeneity within Black African populations when primary studies do not report detailed sociodemographic information.

### 3.2 Conclusion

This systematic review protocol establishes a transparent and reproducible approach to synthesizing qualitative evidence on the maternal healthcare experiences of Black African women in the United Kingdom. By examining cultural, interpersonal, structural, and service‐level factors, the completed review will address a gap created by broader evidence syntheses that aggregate ethnically diverse populations.

## Author Contributions

Irene Devine Dzirasa conceptualized the systematic review and drafted the protocol. Nasima Akhter, Esther Mireku, Modestus Okonkwo, and Lawrence Achilles Nnyanzic critically reviewed the manuscript and provided feedback. All authors read and approved the final version of the manuscript and agree to be accountable for their contributions to the work. Irene Devine Dzirasa affirms that this manuscript is an accurate and transparent account of the planned systematic review and that any deviations from the registered protocol will be documented and explained in the completed review.

## Funding

The authors have nothing to report.

## Ethics Statement

The authors have nothing to report.

## Consent

The authors have nothing to report.

## Conflicts of Interest

The authors declare no conflicts of interest.

## Supporting information


Supporting File

## Data Availability

Data sharing is not applicable to this article as no data sets were generated or analyzed during the current study.
