# Supplementary material for: Factors Influencing Maternal Healthcare Experiences Among Black African Women in the United Kingdom: A Systematic Review Protocol
Source: Health Sci Rep. 2026 Oct 28;9(11):e73450. doi: 10.1002/hsr2.73450 (PMC13715068; doi:10.1002/hsr2.73450)
Supplement: Supplementary file 1 [file HSR2-9-e73450-s001.docx]

**Appendices**

**Table I: Inclusion and exclusion criteria**

| **Criterion** | **Inclusion Criteria** | **Exclusion Criteria** |
| --- | --- | --- |
| **Study Designs** | • Qualitative studies (e.g., phenomenological, grounded theory, ethnography, narrative inquiry)    • Mixed-methods studies containing a substantial, extractable qualitative component | Purely quantitative studies    • Review articles, systematic reviews, meta-analyses, and literature syntheses    • Non-empirical works (editorials, commentaries, opinion pieces, conference abstracts) |
| **Population Characteristics** | • Women identifying as Black African, including Black British women of African descent  • Women who have accessed UK maternity care (antenatal, intrapartum, or postnatal) | • Women from other ethnic groups (e.g., South Asian, East Asian, White minority groups)  • Studies of broadly defined “ethnic minority” or “BAME” populations where findings for Black African women cannot be isolated or extracted separately. |
| **Publication Types** | • Peer-reviewed journal articles  • Eligible grey literature containing primary qualitative data (e.g., national or organisational reports, charity inquiries, or institutional reports) to mitigate publication bias | • Academic books, textbooks, blog posts, and theses/dissertations  • Non-empirical publications, including editorials, commentaries, opinion pieces, conference abstracts/proceedings, reviews, systematic reviews, and meta-analyses |
| Publication Date | Studies published between 2015 and 2025. | Studies published before 2015 or after 2025. |
| **Language Restrictions** | Studies published in full text in English. | Studies published in languages other than English. |
| **Geographical Scope** | • Research conducted exclusively in the United Kingdom (England, Scotland, Wales, and Northern Ireland) | Studies conducted outside the United Kingdom. |
| **Outcome Measures** | • Lived experiences, perceptions, and beliefs    • Identified structural barriers (e.g., administrative, cultural, financial, legal)    • Facilitators to care engagement (e.g., specialist midwifery, continuity of care models) | Clinical outcomes only (e.g., mortality, pre-eclampsia, or neonatal Apgar scores) without qualitative exploration of experiences. |

**Table II. Complete Search strings for each database**

| **Database** | **Complete Search Query / Syntax** | Applied Limits & Restrictions |
| --- | --- | --- |
| **MEDLINE** *(Ovid)* | 1. exp Black People/ OR exp Africa/ OR African/    2. ("Black African*" OR "African descent" OR "Somali*" OR "Ghanaian*" OR "Nigerian*" OR "Black woman" OR "Black women").ti,ab.    3. 1 OR 2    4. exp Maternal Health Services/ OR exp Prenatal Care/ OR exp Postnatal Care/ OR exp Parturition/    5. ("maternal health*" OR "antenatal care" OR "prenatal care" OR "intrapartum care" OR "postnatal care" OR "postpartum" OR "childbirth" OR "obstetric*").ti,ab.    6. 4 OR 5    7. exp Patients' Rooms/ OR exp "Attitude of Health Personnel"/ OR "health equity"/    8. (experience* OR barrier* OR facilitator* OR challenge* OR perception* OR obstacle* OR enabler* OR perspective* OR view* OR belief*).ti,ab.    9. 7 OR 8    10. exp United Kingdom/ OR Great Britain/ OR England/ OR Scotland/ OR Wales/ OR Northern Ireland/    11. ("United Kingdom" OR "UK" OR "Britain" OR "England" OR "Scotland" OR "Wales" OR "Northern Ireland" OR "NHS").ti,ab.    12. 10 OR 11    13. 3 AND 6 AND 9 AND 12 | • Date range: 2015–2025  • Language: English  • Study design filter: Qualitative/mixed-methods (Ovid Qualitative Filter applied) |
| **CINAHL** *(EBSCO)* | S1. (MH "Blacks+") OR (MH "Black African +")    S2. TI ( "Black African*" OR "African descent" OR "Somali*" OR "Ghanaian*" OR "Nigerian*" OR "Black woman" OR "Black women" ) OR AB ( "Black African*" OR "African descent" OR "Somali*" OR "Ghanaian*" OR "Nigerian*" OR "Black woman" OR "Black women" )    S3. S1 OR S2    S4. (MH "Maternal Health Services+") OR (MH "Antenatal Care+") OR (MH "Postnatal Care+") OR (MH "Childbirth+")    S5. TI ( "maternal health*" OR "antenatal care" OR "prenatal care" OR "intrapartum care" OR "postnatal care" OR "postpartum" OR "childbirth" OR "obstetric*" ) OR AB ( "maternal health*" OR "antenatal care" OR "prenatal care" OR "intrapartum care" OR "postnatal care" OR "postpartum" OR "childbirth" OR "obstetric*" )    S6. S4 OR S5    S7. TI ( experience* OR barrier* OR facilitator* OR challenge* OR perception* OR obstacle* OR enabler* OR perspective* OR view* OR belief* ) OR AB ( experience* OR barrier* OR facilitator* OR challenge* OR perception* OR obstacle* OR enabler* OR perspective* OR view* OR belief* )    S8. (MH "United Kingdom+") OR TI ( "United Kingdom" OR "UK" OR "Britain" OR "England" OR "Scotland" OR "Wales" OR "Northern Ireland" OR "NHS" ) OR AB ( "United Kingdom" OR "UK" OR "Britain" OR "England" OR "Scotland" OR "Wales" OR "Northern Ireland" OR "NHS" )    S9. S3 AND S6 AND S7 AND S8 | • Date range: 2015–2025  • Language: English  • Study design limit: Clinical Queries: "Qualitative" |
| **EMBASE** *(Ovid)* | 1. exp black person/ OR exp African/    2. ("Black African*" OR "African descent" OR "Somali*" OR "Ghanaian*" OR "Nigerian*" OR "Black woman" OR "Black women").ti,ab.    3. 1 OR 2    4. exp maternal care/ OR exp prenatal care/ OR exp postnatal care/ OR exp childbirth/    5. ("maternal health*" OR "antenatal care" OR "prenatal care" OR "intrapartum care" OR "postnatal care" OR "postpartum" OR "childbirth" OR "obstetric*").ti,ab.    6. 4 OR 5    7. ("experience*" OR "barrier*" OR "facilitator*" OR "challenge*" OR "perception*" OR "obstacle*" OR "enabler*" OR "perspective*" OR "view*" OR "belief*").ti,ab.    8. exp United Kingdom/ OR exp Great Britain/    9. ("United Kingdom" OR "UK" OR "Britain" OR "England" OR "Scotland" OR "Wales" OR "Northern Ireland" OR "NHS").ti,ab.    10. 8 OR 9    11. 3 AND 6 AND 7 AND 10 | • Date range: 2015–2025  • Language: English  • Study design filter: Qualitative/mixed-methods |

**Table III. Data extraction form**

| **Citation** | Full reference of the article, including author name(s), year, title, and source. |
| --- | --- |
| Country and setting of study |  |
| **Date of extraction** |  |
| **Reviewer name** |  |
| **Purpose/objectives of study** | Purpose/objectives of the study as stated by the study authors. |
| Study design  - Type of qualitative study or mixed-methods design - Duration of study | Type of qualitative study or mixed-methods study with an extractable qualitative component, together with the duration of the study. |
| Study population  - Number of participants - Type/characteristics of participants - Relevant sociodemographic characteristics | Description of the study sample, including sample size and relevant participant characteristics. |
| Study outcomes/findings  - Experiences - Barriers - Facilitators - Challenges - Enablers | Findings relevant to maternal healthcare experiences, barriers, facilitators, challenges, and enablers. |
| **Results of the study** | Details of the study findings relevant to the review question. |
| Study limitations | Limitations reported by the study authors and/or identified during appraisal. |

**Table IV. The Critical Appraisal Skills Programme Qualitative (CASP) checklist**

| CASP qualitative appraisal question | **Yes** | Can’t tell | **No** | **Hint** |
| --- | --- | --- | --- | --- |
| 1. Was there a clear statement of the aims of the research? |  |  |  | Consider whether the study clearly states its aim, rationale, and relevance. |
| 2. Is a qualitative methodology appropriate? |  |  |  | Consider whether a qualitative methodology is appropriate for the research question. |
| 3. Was the research design appropriate to address the aims of the research? |  |  |  | Consider whether the authors justify the research design in relation to the study aims. |
| 4.Was the recruitment strategy appropriate to the aims of the research? |  |  |  | Consider whether the participant-selection and recruitment strategy is appropriate and adequately justified. |
| 5.Was the data collected in a way that addressed the research issue? |  |  |  | Consider whether the data-collection methods are justified and appropriate, including discussion of saturation where relevant. |
| 6. Has the relationship between researcher and participants been adequately considered? |  |  |  | Consider whether the researchers adequately address reflexivity and potential researcher influence on the research question, data collection, and study setting. |
| 7. Have ethical issues been  taken into consideration? |  |  |  | Consider the ethical issues relevant to the study and how they were addressed. |
| 8. Was the data analysis  sufficiently rigorous? |  |  |  | Consider whether the analysis is sufficiently rigorous and whether potential bias in analysis and presentation is addressed. |
| 9. Is there a clear statement of findings? |  |  |  | Consider whether the findings are clearly stated, sufficiently supported, and critically discussed. |
| 10. How valuable is the research? |  |  |  | Consider the contribution of the research to existing knowledge, practice, or policy and the transferability of the findings. |
